# Does migration ‘pay off’ for foreign-born migrant health workers? An exploratory analysis using the global WageIndicator dataset

**DOI:** 10.1186/s12960-016-0136-5

**Published:** 2016-06-24

**Authors:** Daniel H. de Vries, Stephanie Steinmetz, Kea G. Tijdens

**Affiliations:** Department of Anthropology, University of Amsterdam, Amsterdam, The Netherlands; Department of Sociology, University of Amsterdam, Amsterdam, The Netherlands; Amsterdam Institute for Advanced Labor Studies (AIAS), University of Amsterdam, Amsterdam, The Netherlands

**Keywords:** Migration, Health worker, Routes, Drivers, Discrimination, Wages, WageIndicator

## Abstract

**Background:**

This study used the global WageIndicator web survey to answer the following research questions: (RQ1) What are the migration patterns of health workers? (RQ2) What are the personal and occupational drivers of migration? (RQ3) Are foreign-born migrant health workers discriminated against in their destination countries?

**Methods:**

Of the unweighted data collected in 2006–2014 from health workers aged 15–64 in paid employment, 7.9 % were on migrants (*N* = 44,394; 36 countries). To answer RQ1, binary logistic regression models were applied to the full sample. To answer RQ2, binary logistic regression was used to compare data on migrants with that on native respondents from the same source countries, a condition met by only four African countries (*N* = 890) and five Latin American countries (*N* = 6356). To answer RQ3, a multilevel analysis was applied to the full sample to take into account the nested structure of the data (*N* = 33,765 individual observations nested within 31 countries).

**Results:**

RQ1: 57 % migrated to a country where the same language is spoken, 33 % migrated to neighbouring countries and 21 % migrated to former colonizing countries. Women and nurses migrated to neighbouring countries, nurses and older and highly educated workers to former colonizing countries and highly educated health workers and medical doctors to countries that have a language match. RQ2: In the African countries, nurses more often out-migrated compared to other health workers; in the Latin American countries, this is the case for doctors. Out-migrated health workers earn more and work fewer hours than comparable workers in source countries, but only Latin American health workers reported a higher level of life satisfaction. RQ3: We did not detect discrimination against migrants with respect to wages and occupational status. However, there seems to be a small wage premium for the group of migrants in other healthcare occupations. Except doctors, migrant health workers reported a lower level of life satisfaction.

**Conclusions:**

Migration generally seems to ‘pay off’ in terms of work and labour conditions, although accrued benefits are not equal for all cadres, regions and routes. Because the *WageIndicator* survey is a voluntary survey, these findings are exploratory rather than representative.

**Electronic supplementary material:**

The online version of this article (doi:10.1186/s12960-016-0136-5) contains supplementary material, which is available to authorized users.

## Background

### Study aim

The chronic worldwide shortage of some 2.4 million physicians, nurses and midwives, and 2 million pharmacists and other paramedical workers, has in the past two decades led to an increase in the international migration of human resources for health [1]. Hagopian et al. estimated that the number of physicians migrating to the United States of America from Nigeria and Ghana increased by more than 1000 % between 1981 and 2002 [2]. Since the 1979 landmark study by Meja et al., concern with this flow has focused on the impact of health worker brain-drain for source (sending) countries, the commercialization of migratory routes and the ethics of international recruitment (e.g. training cost shouldered by low income countries) [3–5]. These concerns have overshadowed the question whether foreign-born migrant health workers are actually better off outside their own countries [6–8], probably because benefits are often presumed to be self-evident.

Research on the net benefits of international migration for health workers, however, is predominantly based on generalized and anecdotal information, with statistics comparing only a handful of countries on a limited number of variables [9]. To establish whether migration ‘pays off’ for foreign-born health workers, micro-level data from source and destination countries are required. Not only are data needed on experiences in the destination country for different cadres regarding a number of job-related factors but these data also have to be evaluated relative to the conditions of peers in source countries [10]. Whereas discrimination analyses can still rely on data on one or a few destination countries, analyses of global migration require micro-data from a large number of countries. Ideally, these analyses require representative multi-country survey data, but such surveys are available only to a limited extent. For example, the World Values Survey database does not contain data on respondents’ earnings—a main driver of migration—or country of birth.

In this study, we relied on the data of the non-probability-based multi-country WageIndicator web survey on work and wages to answer the question whether migration pays off for foreign-born health workers. We defined ‘pay off’ in terms of wages, working hours, and life and job satisfaction compared to peers in the country of birth. We also explored potential wage discrimination compared to peers in the country of destination. In addition, we used the dataset to explore the migratory routes taken. Although the data are not representative and these results can only be perceived as explorative, the clear benefit of using the WageIndicator is that it provides detailed data on all variables needed to model and explore global patterns of human resources for health [11, 12].

### The benefits and challenges of migration

Migrant health workers are thought to benefit from migration as a result of increased remuneration and benefits [10, 13–15], professional development and continuing education [7, 10, 16–18] and better working conditions, including flexible scheduling and shift rotation, safe working environments, team support and job security [10, 19]. Migrants are said to have more autonomy and involvement in decision making [10] and a lighter workload [20]. In addition, migrants enhance their professional ability to provide quality care and a commitment to excellence, and feel valued as health professionals by management and peers [10]. Finally, migrants are said to have an enhanced quality of life and diverse cultural experiences [5, 19, 20], including opportunities to join previously migrated family members [20] and, last but not least, the ability to send significant remittances back home [19, 21].

Despite this, migration also has a darker side. For example, the British Royal College of Nurses reported that a common experience among overseas nurses is a lack of recognition of their skills and previous experience, leading to a feeling that their competence as nurses is being questioned [22]. Tregunno noted that migration is not uncommonly experienced as a ‘U-turn’ from clinical expert to cultural novice when health workers enter practice in their adopted country [23]. These experiences may lead to relatively more code violations resulting from difficulties in the delivery of safe and ethical care [23, 24], problems dealing with more demanding patients and patients’ families (e.g. informed consent), and language issues [25, 26]. Many migrant health workers complain that managers and colleagues behave inappropriately towards them and report incidents of bullying, racism and a lack of professionalism [9, 27, 28]. Aboderin documented the way in which migrant nurses in the UK perceive a lack of respect and pressure to perform menial tasks from colleagues who typically view them as ‘economic migrants’, while being subjected to more intensive controlling supervision than their colleagues [21].

Nurses and women are particularly affected by a lack of management once they reach their destination sites [29]. In the context of the popular UK migration path, Adhikari noted that ‘while government attention has been focused on managing the flow and being an ethical recruiter, the migrant nurses who are already in the UK have been completely ignored’ [27]. Because of their migrant status, women caregivers in particular are at high risk of abuse and exploitation [30, 31], including sexual advances from patients, patients’ family members and doctors while working [28]. Migrants also experience discriminatory labour rights compared to locals [32], including poor pay, discriminatory conditions of service and in some cases racism [9, 26]. As result of licensing problems, many are pushed into the private sector [27] and are typically working below their skill set [25, 29, 31, 33, 34] and tied to a job by work permits that limit their ability to look for more suitable positions. Migrant nurses might find that they have been promised more by recruiters than they actually receive, and many recruiters fail to fully explain the higher cost of living in destination countries and the effect it has on the promised salary [26]. Failure to meet their hopes and expectations causes disappointment and disillusionment, which may lead many to consider onward migration [29]. Nurses often end up in vulnerable and inappropriate employment, especially when expensive migration brokers are involved. New commercial entry routes, visa uncertainties, loans and other financial challenges can make it harder for nurses to leave [27, 35]. This can cause emotional distress, feelings of hopelessness and depression; in some cases, it has led to suicide [28].

Considering the potential negative outcomes of migration, one expectation may be that only the most elite within source countries may end up enjoying a positive experience. McElmurry noted that recipient countries are able to select ‘the best of the best’ from source countries [26]. Findings indicate that those who migrate indeed already have a distinct or privileged social status [10, 21, 32]; they are often young, up-and-coming professionals in whom heavy investments have been made [36, 37]. Two studies have reported that a higher proportion of male nurses had either migrated [38] or intended to migrate [10] compared to female nurses. Furthermore, the benefits that migrants accrue are relative to the level of development of source countries [39] and the level of marginality of regions within those countries (e.g. rural areas) [36]. For example, Diallo noted that source countries that have a sufficient number of health workers mainly benefit from remittances, while source countries that are losing workers and are already suffering from shortages experience a further decline in working conditions and quality of care [40].

The migratory routes that these migrants typically take are not entirely random but are influenced by historical and colonial ties between countries that facilitate migration and buffer against negative influences [28]. However, some have argued that as a result of globalization, these ties are loosening as destination countries become more utilitarian in encouraging migration primarily on the basis of economic requirements [7, 41]. This has shifted attention to the influence of more complex global care chains that include, for example, networks of colleagues already abroad [9, 20, 28]. Migration is often facilitated by mutually beneficial cross-border exchanges between neighbouring countries. This helps to overcome cultural and institutional differences, such as language differences or difficulties harmonizing titles and degrees [14, 42]. Nurse migration has been described as a ‘carousel’ rather than a simple south to north flow, including movement from the public to the private sector, from rural to urban locations and between multiple transnational destinations [43].

### Research questions and hypotheses

Based on the above review of factors that may influence successful migration, three main research questions were defined:

RQ1: What are the migration patterns of health workers? Here, we identified whether migration patterns are predominantly shaped by:

- Similar languages in the source and destination countries;
- Source and destination countries bordering each other; and/or
- Source and destination countries having a previous colonial relationship.

RQ2: What are the personal and occupational drivers of migration of health workers? Here, we explored:

- Whether the most qualified health workers migrate, by comparing the personal characteristics of out-migrated health workers with those of health workers in their respective source countries; and
- Whether migration pays off in terms of wages, working hours, and life and job satisfaction, by comparing the characteristics of out-migrated health workers with those of health workers in their respective source countries.

RQ3: Are foreign-born migrant health workers discriminated against in their destination countries? Here, we investigated:

- Whether these migrants suffer downward mobility, by exploring whether they end up in lower paid healthcare occupations even when they have the same qualifications as native health workers;
- Whether these migrants suffer pay discrimination, by exploring whether they receive lower wages or have longer working hours than native health workers in the same healthcare occupations; and
- Whether these migrants report an overall lower level of work and life satisfaction than native health workers.

## Methods

### Data

The WageIndicator web survey is posted continuously on national WageIndicator websites (www.wageindicator.org). The first WageIndicator website was launched in the Netherlands in 2001. Today, it is operational in 85 countries and receives millions of visitors (25 million in 2014). The websites provide job-related content, information on labour laws and minimum wages, wages of celebrities and a free ‘salary check’ (i.e. average wages based on the web survey). In reciprocation for this free information, web visitors are asked to complete a questionnaire and are encouraged to do so by being offered the chance to win a lottery. The questionnaire is posted continuously on the national websites and uses the same survey questions across countries. The questionnaire is in the national language(s), adapted to the specific country, and covers a wide range of work- and wage-related subjects as well as socio-demographics. The number of respondents in each country depends on the number of web visitors and thus varies substantially across countries.

To obtain a sufficient number of observations, we pooled the survey data collected between 2006 and 2014. All analyses were controlled for year of survey. We restricted the dataset to workers in healthcare, defined using the International Standard Classification of Occupations (ISCO–08). This resulted in a list of 20 occupations (see Additional file 1). We further restricted the dataset to workers in healthcare and social services (NACE industry classification sector Q), plus those working in a healthcare occupation in education, the public sector and commercial industries (NACE sectors M, O, P). The reason for this broad scope is that people in healthcare occupations do not only work in the healthcare sector. For example, community health workers are predominantly employed in the public sector and health education professionals in the education sector. In this article, we use the term ‘healthcare occupations’ for the selected group of workers.

We then limited our sample to respondents in paid employment, either as employees, temporary agency workers, self-employed individuals or apprentices, and to respondents aged 15–64 years. Further, we selected respondents who provided a valid answer to the survey question ‘Were you born in [COUNTRY OF SURVEY]?’ (Y/N), which allowed us to create a binary variable for migrant versus native workers. If not born in the country of survey (country of residence), a follow-up question asked ‘In which country were you born?’ Unfortunately, no information was available on countries of residence other than county of birth and current country of residence, or on the length of residency in the country of birth. Finally, given that sample sizes varied widely across countries, only countries with at least 50 valid observations were included.

After these selections, a total number of observations *N* = 44,394 from 36 countries remained (see Additional file 2 (a and b) for sample sizes broken down by country and year of survey). In this sample, 7.9 % of the respondents were classified as migrants. The country of birth is missing for a minor group of these migrants, but for 6.6 % the country of birth is known, totalling 164 source countries (*N* = 2931). To answer the three research questions, we made different selections from the sample; this is explained in more detail in the ‘Operationalization and analytical strategy’ section.

With respect to the problem of sample bias due to the voluntary nature of the data collection, we addressed the issue of whether some type of post-survey adjustment could to some extent correct for biases related to core sociodemographic variables [44, 45]. In the case of the WageIndicator data, several studies have shown that most web samples deviated to some extent from representative reference samples with regard to the common variables of age, gender and education [46–50]. It has also been shown that the sample bias differs tremendously across countries, with higher selectivity in countries with lower Internet penetration rates and growth [51].

To deal with this problem, we considered various adjustment techniques (e.g. post-stratification weighting and propensity score adjustment), but also here the results remain inconclusive regarding the efficiency of the weights. For instance, in a previous study focusing on three European countries, we investigated the bias in the healthcare labour force and the possibility of simple post-stratification weights by comparing our sample with Eurostat’s labour force data for the years 2008 to 2012 (NLD until 2011) [52]. The comparison showed that, in all countries and in all years, the age group 20–49 was overrepresented in the web survey for both sexes, whereas the age group 50–59 was underrepresented. Moreover, the implementation of proportional weights did not change the outcome greatly.

Due to the rather inconclusive results regarding the efficiency of post-survey adjustment methods, and the fact that we lacked representative reference surveys that could be used for weighting for all included countries, we decided to use the unweighted data and consider the results as exploratory rather than representative.

### Operationalization and analytical strategy

The purpose of research question 1 was to identify whether migration patterns are shaped by language matches, neighbouring countries or former colonizing countries. For this, the full sample was restricted to migrants with a known country of birth (*N* = 2931). Three binary dependent variables were created to compare the 164 source countries and the 36 survey countries with respect to: (a) their native language(s) and the lingua franca of both countries, indicating a language match in the source and destination country (1 = yes, 0 = no); (b) whether source and destination country border each other (using the lists of bordering countries in the CIA World Factbook; 1 = yes, 0 = no); and (c) whether source and destination country previously had a colonial relationship (using Wikipedia and historical review papers, previous colonial rulers were identified for source countries, 1 = yes, 0 = no). Applying binary logistic regression models, we controlled for gender (ref. male), age (in years), education (based on the internationally recognized standard classification (ISCED 1997) differentiating three categories, i.e. ‘low’ (ISCED 0–2), ‘medium’ (ISCED 3–4, ref.) and ‘high’ (ISCED 5–6)) and two healthcare occupations, namely doctor and nurse (ref. ‘other healthcare occupations’). These two occupations were derived from the list of 20 healthcare occupations (doctors = 1 Medical Doctors; nurses = 2 Nursing & Midwifery Professionals plus 13 Nurses & Midwifery Associate Professionals). Slightly more than 40 observations had one or more missing values within these variables, thereby reducing our sample of migrants to *N* = 2888. Additional file 3 shows which job titles are included to classify doctors and nurses according to the ISCO–08 classification.

The purpose of research question 2 was to identify whether the most qualified migrate, by comparing the personal characteristics of migrants with those of the workers in their respective source countries. This analysis required data from countries that had a sufficient number of out-migrated workers and a sufficient number of native workers (a minimum of 10 was required for both types of workers). We identified which of the 164 source countries had the largest groups of migrants. For the analysis, we selected the data on the migrants originating in these countries plus the data on the native respondents in these countries. The condition of 10 observations for both migrants and natives was jointly met by four African countries—Angola, Kenya, South Africa and Zimbabwe—with a total of 890 observations (16 % are out-migrated workers), and five Latin American countries—Argentina, Brazil, Chile, Colombia and Mexico—with 6356 observations (3 % are out-migrated workers). In the next step, we explored whether migration pays off with respect to wages (log-hourly wages), working hours (in 5-h groups) and level of life satisfaction (on a scale from 1 = dissatisfied to 10 = satisfied). s The dependent variable was outmigration (1 = yes, 0 = no). Applying binary logistic regression models, we controlled the analysis again for gender, age, education, the purchasing power (PP) standardized hourly wages, and doctors and nurses. Excluding the cases with missing values on either the dependent or the independent variables, the sample sizes varied per analysis, notably for wages (*N* = 688 for African countries/*N* = 5109 for Latin American countries); for working hours (*N* = 827 for African countries/*N* = 6274 for Latin American countries); and for level of life satisfaction (*N* = 796 for African countries/*N* = 6093 for Latin American countries). Note that we restricted the sample to workers in healthcare occupations. We do not know the occupation before migrating. Therefore, the sample does not include out-migrating workers who worked in a healthcare occupation before migrating, but after migrating did not find a job or did not work in healthcare.

The purpose of research question 3 was to identify whether migrants are discriminated against in their destination countries. In this context, we explored three types of discrimination, namely downward mobility (the International Socio-Economic Index (ISEI), scale from 16 = lowest status to 89 = highest status), lower wages (the PP-standardized hourly wages) and lower level of life satisfaction (scale from 1 = dissatisfied to 10 = satisfied). Applying multilevel analyses (random intercept models), which took into account the nested structure of the data, we controlled again for gender, age, age squared, education, as well as the occupational groups of doctors and nurses. In this analysis, we used the full sample; hence, we used the data from the country of residence for the native workers and the migrant workers in these countries. However, due to missing values, five countries (Angola, Estonia, Guatemala, Kenya and Egypt) had to be excluded, as their total sample size fell below 50 valid observations; consequently, the final sample consisted of *N* = 33,765 observations at the individual level, nested within 31 countries. Note that no data were available on age at migration or education in the country of birth (only education in the destination country). If respondents were educated in their country of birth, the survey instructed them to tick an equivalent education in the destination country.

## Results

### Migration patterns (RQ1)

In the 36 destination countries, on average almost 10 % of the respondents were migrants, but the proportion varied considerably across countries, as shown by column 3 in Additional file 2. For instance, in Angola, Mozambique, Denmark, the United Kingdom and the United States, approximately 20–40 % of health workers are migrants, whereas in Brazil, China, Colombia, Estonia, Finland, Guatemala, Indonesia, South Korea, Mexico, Poland, Slovakia and Egypt, hardly any migrants are health workers. The migrants in the 36 countries surveyed originate in a total of 164 source countries. See columns 5–9 in Additional file 2 for the five most frequently mentioned source countries per destination country.

Starting with a description of different migration patterns, Table 1 shows that the majority of migrants (57 % in our sample) migrated to a country where the same language is spoken. In particular, there are very high levels of migration between the Portuguese-speaking countries of Angola, Mozambique, Brazil and Portugal (see Additional file 2). To a somewhat lesser extent, migration occurred between neighbouring countries: one third of the study participants migrated to a neighbouring country (see Table 1). Finally, migration is also shaped by previous colonial relationships between the source and destination countries: Table 1 shows that one in five migrants migrated to a former colonizing country.

**Table 1:** Migrants, distributed over eight categories of source versus destination country combinations

	Different language (%)	Same language (%)	Total (%)
No colonizer—not-neighbouring country	31	16	47
No colonizer—neighbouring country	7	25	32
Colonizer—not-neighbouring country	5	15	20
Colonizer—neighbouring country	0	1	1
Total	43	57	100

Source: WageIndicator 2006–2014, selection migrants in healthcare occupations with identified country of birth. *N* = 2888, Chi square = 261.05 (*p* = .000)

Turning to multiple analyses in order to test whether these patterns hold when controlled for personal characteristics, the first panel in Table 2 shows that the odds of migrating to a neighbouring country are six times higher for those who migrated to a country where the same language is spoken. Moreover, in comparison to men, women have a 39 % higher chance of migrating to a neighbouring country. In comparison to medium educated individuals, low educated ones have a 44 % lower chance of migrating to a neighbouring country. Occupation does not impact migrating to a neighbouring country.

**Table 2:** Likelihood of migration to a neighbouring country (1 = yes, 0 = no), to a country with the same language (1 = yes, 0 = no), and to a former colonizer (1 = yes, 0 = no), standard errors in brackets

	DV neighbour country	DV same language	DV former colonizer
	Exp(B)	Exp(B)	Exp(B)
Neighbour country		6.16***	0.06***
		(0.1)	(0.18)
Same language	6.00***		8.36***
	(0.10)		(0.12)
Former colonizer	0.06***	9.36***	
	(0.18)	(0.12)	
Female	1.39***	1.06	1.18
	(0.10)	(0.10)	(0.13)
Age	1.00	1.00	1.03***
	(0.00)	(0.00)	(0.00)
High education	0.92	1.59***	0.66***
	(0.11)	(0.10)	(0.13)
Low education	0.56***	1.14	1.07
	(0.20)	(0.17)	(0.20)
Nurse	1.24*	0.89	1.36**
	(0.11)	(0.10)	(0.13)
Med. doctor	1.03	1.52***	1.30
	(0.14)	(0.14)	(0.17)
Constant	0.11***	0.14***	0.11***
	(0.29)	(0.26)	(0.31)
Year controlled 2006–2014	Yes	Yes	Yes
Chi square	863.52, df(16) ***	793.93, df(16) ***	806.80, df(16) ***
−2 Log likelihood	2831.96	3183.04	2162.71

Source: WageIndicator 2006–2014, selection migrants in healthcare occupations with identified country of birth. *N* = 2904, reference categories: middle education, all other healthcare occupations; year 2006

*Significant at 10 %; **significant at 5 %; ***significant at 1 %

The second panel shows that the odds of migrating to a country with a language match are six times higher for those migrating from a neighbouring country and nine times higher for those migrating from a former colonizing country. No significant effects are noticed for gender or age. For education, a significant effect can be observed for more highly educated people, indicating that in comparison to medium educated individuals, highly educated people have a 59 % higher chance of migrating to a country with a language match. Significant effects can also be observed for occupations. In comparison to other healthcare occupations, for doctors, the odds of migrating to a country with a language match are 52 % higher, whereas no significant results are found for nurses.

Finally, the third panel in Table 2 shows the results for migrating to a former colonizing country. While the odds of migrating to a former colonizing country are 94 % lower for those who migrated from a neighbouring country, they are eight times higher for those who migrated from a country where the same language is spoken. Moreover, the results indicate that with each increase in age by 1 year, the odds of migrating to a former colonizing country are 3 % higher. In comparison to medium educated people, highly educated individuals have a 34 % lower chance of migrating to a former colonizing country. Finally, in comparison to other healthcare occupations, the odds of nurses migrating to a former colonizing country are 36 % higher.

In sum, migration has been shaped by language matches, neighbouring countries and former colonizing countries, but the migrants’ characteristics differ. Whereas female migrants migrated more to neighbouring countries, age only shows a significant impact for former colonizing countries. The results further indicate that higher education motivates migration to a country where the same language is spoken, whereas the reverse holds for migration to former colonizing countries. Finally, with respect to the health workforce, nurses migrate more to neighbouring countries and former colonizing countries, whereas doctors are much more likely to migrate to countries that have a language match.

### Drivers of migration

#### Which workers migrate? (RQ2)

To establish whether the ‘best’ workers migrate, we compared the outgoing migrants with non-migrating native workers from the same source countries for four African and five Latin American countries. The descriptive bivariate analysis in Additional file 4 shows that the out-migrated healthcare workers are more often female in the Latin American countries, whereas no difference is found for the African countries. They are slightly older in both the African and the Latin American countries. No difference exists concerning the percentages of highly educated workers, but the percentage of low educated workers is higher for those out-migrated in the Latin American countries. Finally, both doctors and nurses (except the nurses in the Latin American countries) have more often migrated out of the country.

Using a multiple approach, Table 3 shows that the bivariate findings for gender are confirmed. The odds ratio of outmigration increases by more than 40 % for women in the Latin American countries. The bivariate findings for age are not confirmed, as age does not have a significant effect on outmigration. The bivariate findings for education are confirmed. Education has no significant effect on outmigration in the African countries, but in the Latin American countries, the odds ratio increases five times for the low educated healthcare workers. The bivariate findings for nurses and doctors are partly confirmed. In the case of the four African countries, no significant effect was found for doctors, but the odds ratio almost doubles for nurses compared to other healthcare occupations. In the Latin American countries, the reverse holds. The bivariate results are confirmed for the doctors, with the odds ratio nearly doubling. For nurses, however, the odds ratio for outmigration is not significantly different from that of the other healthcare occupations.

**Table 3:** Likelihood of migrating out of the country for all persons born in the country, robust standard errors in brackets

	4 African countries	5 LATAM countries
	Exp(B)	Exp(B)
Female	0.83	1.42**
	(0.21)	(0.24)
Age	1.02	1.01
	(0.01)	(0.00)
High education	0.82	1.22
	(0.23)	(0.25)
Low education	1.00	5.94***
	(0.72)	(2.34)
Nurse	1.71*	1.00
	(0.46)	(0.23)
Med. doctor	1.52	2.02***
	(0.68)	(0.43)
Constant	9.63***	0.01***
	(7.09)	(0.005)
Year controlled 2006–2014	Yes	Yes
Wald Chi square	88.72, df(13)***	77.44, df(13)***
−2 Log likelihood	−280.17	−796.22
Number	890	6356

Source: WageIndicator 2006–2014, selection health workers born in four African countries (Angola, Kenya, South Africa and Zimbabwe) and in five Latin American countries (Argentina, Brazil, Chile, Colombia and Mexico). Reference categories: middle education, all other healthcare occupations, year 2006–2007

*Significant at 10 %; **significant at 5 %; ***significant at 1 %

#### Does migration ‘pay off’ in wages?

To find out whether migration pays off in different ways (wages, working hours and level of life satisfaction), we reused the data on the four African and the five Latin American countries. Starting with the analyses for wages, Table 4 model 1 shows that for both country groups, those who out-migrated have substantially higher wages (51 % for African and 65 % for Latin American countries) compared to those who remained in the country. Note that the income of self-employed workers in the sample was recoded to hourly wages. This effect remains when controlling for the variables age, gender, education, organization size and occupation, which also indicates that migration definitely pays off. In addition, model 2 shows that, as expected, on both continents wages increase significantly with age, higher education and a larger organization, and for doctors, whereas wages decrease significantly for women. No significant wage effect was found for nurses in both country groups. Finally, when looking at the effect of outmigration for particular health occupations, model 3 shows that particularly for nurses in the Latin American countries and the doctors, the positive effect of outmigration is reduced significantly.

**Table 4:** OLS regression for log gross hourly wage, corrected for purchasing power parity (PPP), unstandardized coefficients, standard error in brackets

	4 African countries			5 Latin American countries		
	M1	M2	M3	M1	M2	M3
(Constant)	2.80***	0.56	0.55	2.06***	0.05	0.07
	(0.19)	(0.44)	(0.44)	(0.38)	(0.37)	(0.38)
Migrated out of country	0.51***	0.48***	0.46***	0.65***	0.55***	0.75***
	(0.11)	(0.10)	(0.11)	(0.09)	(0.08)	(0.10)
Female		−0.11*	−0.11*		−0.16***	−0.16***
		(0.06)	(0.06)		(0.03)	(0.03)
Age		0.06***	0.06***		0.08***	0.07***
		(0.02)	(0.02)		(0.01)	(0.01)
Age_sq		0.00*	0.00*		0.00***	0.00***
		(0.00)	(0.00)		(0.00)	(0.00)
High education		0.56***	0.56***		0.49***	0.49***
		(0.07)	(0.07)		(0.03)	(0.03)
Low education		−0.01	−0.10		0.09	0.07
		(0.21)	(0.21)		(0.11)	(0.11)
Log firm size		0.20***	0.20***		0.09***	0.09***
		(0.04)	(0.04)		(0.02)	(0.02)
Nurse		−0.10	−0.14		0.05	0.06
		(0.09)	(0.10)		(0.04)	(0.04)
Med. doctor		0.27**	0.30**		0.54***	0.56***
		(0.13)	(0.14)		(0.04)	(0.04)
Nurse*outmigration			0.15			−0.44*
			(0.20)			(0.23)
Med. doctor*outmigration			−0.08			−0.68***
			(0.29)			(0.20)
Year controlled 2006–2014	Yes	Yes	Yes		Yes	Yes
*R*	0.295	0.554	0.545	0.146	0.472	0.474
Number	736	736	736	5111	5111	5111

Source: WageIndicator 2006–2014, selection health workers born in four African countries (Angola, Kenya, South Africa and Zimbabwe) and in five Latin American countries (Argentina, Brazil, Chile, Colombia and Mexico). Reference categories: middle education, all other healthcare occupations, year 2006

*Significant at 10 %; **significant at 5 %; ***significant at 1 %

#### Shorter working hours for out-migrating workers?

In addition, we wanted to determine whether migration also pays off in terms of working hours; that is, to find out whether out-migrating workers have shorter working hours compared to their peers who remained in the country. The results of the OLS regression presented in Table 5 show that out-migrating health workers have substantially shorter working hours compared to their peers. In African countries, out-migrated workers work 7 h a week less than their peers who remained in the country, and in the Latin American countries they work almost 1.5 h less a week (see model 1). This effect remains significant even when controlling for sociodemographic variables (model 2). The model shows that women work fewer hours than men in African and Latin American countries and that the high educated work fewer hours in the Latin American countries. Model 2 also shows that independent of outmigration, doctors work significantly longer hours compared to other healthcare workers in both the African and the Latin American countries, whereas nurses do so only in the Latin American countries. As for whether the observed reduction in working hours for out-migrants differs among health occupations, the results of model 3 show that outmigration seems to pay off in terms of reduced working hours for nurses in the African countries and for doctors in the Latin American countries.

**Table 5:** OLS regression for working hours per week, unstandardized coefficients, standard error in brackets

	4 African countries			5 Latin American countries		
	M1	M2	M3	M1	M2	M3
(Constant)	46.14***	38.51***	38.88***	27.90***	31.42***	31.23***
	(2.28)	(5.51)	(5.50)	(4.30)	(4.73)	(4.75)
Migrated out of country	−7.02***	−7.21***	−6.19***	−1.40**	−1.38	−0.27
	(1.24)	(1.23)	(1.37)	(0.93	(0.93)	(1.14)
Female		−1.62**	−1.61**		−2.42***	−2.42***
		(0.81)	(0.81)		(0.31)	(0.31)
Age		0.46*	0.48*		−0.01	−0.01
		(0.28)	(0.28)		(0.11)	(0.11)
Age_sq		−0.01*	−0.01**		0.00	0.00
		(0.00)	(0.00)		(0.00)	(0.00)
High education		−0.94	−1.00		−1.89***	−1.91***
		(0.90)	(0.90)		(0.35)	(0.35)
Low education		−1.49	−1.60		1.64	1.62
		(2.69)	(2.68)		(1.37)	(1.37)
Log firm size		0.78*	0.75		0.10	0.10
		(0.47)	(0.47)		(0.20)	(0.20)
Nurse		0.64	2.24*		1.44***	1.49***
		(1.07)	(1.21)		(0.45)	(0.45)
Med. doctor		6.76***	5.72***		1.78***	1.94***
		(1.53)	(1.72)		(0.46)	(0.47)
Nurse*outmigration			−6.43***			−1.83
			(2.45)			(2.69)
Med. doctor*outmigration			3.73			−3.98*
			(3.65)			(2.25)
Year controlled 2006–2014	Yes	Yes	Yes	Yes	Yes	Yes
*R*	0.212	0.291	0.308	0.138	0.193	0.194
Number	884	884	884	6276	6276	6276

Source: WageIndicator 2006–2014, selection health workers born in four African countries (Angola, Kenya, South Africa and Zimbabwe) and in five Latin American countries (Argentina, Brazil, Chile, Colombia and Mexico). Reference categories: middle education, all other healthcare occupations, year 2006

*Significant at 10 %; **significant at 5 %; ***significant at 1 %

#### Higher level of life satisfaction for out-migrating workers?

Lastly, we wanted to know whether migration pays off other than in the form of monetary rewards or better working conditions by looking at the effect of outmigration on level of life satisfaction. The findings (model 1) in Table 6 show that in both the African and the Latin American countries, out-migrated health workers report a significantly higher level of life satisfaction compared to their peers who remained in the country. This effect persists when controlling for other relevant personal characteristics (model 2). In this context it is interesting to note that independent of outmigration, highly educated individuals report a higher level of life satisfaction. In addition, working in a larger organization affects the level of life satisfaction positively in the African countries, and doctors in the Latin American countries report a higher level of life satisfaction compared to those in other healthcare occupations. With respect to whether outmigration pays off for particular health occupations (model 3), the results show that in the African countries out-migrated doctors report a higher level of life satisfaction, whereas in the Latin American countries no significant effect was found.

**Table 6:** OLS regression for satisfaction with life as a whole: 1 = dissatisfied–10 = satisfied, unstandardized coefficients, standard error in brackets

	4 African countries			5 Latin American countries		
	M1	M2	M3	M1	M2	M3
(Constant)	5.19***	4.91***	4.78***	7.46***	7.98***	7.85***
	(0.49)	(1.17)	(1.17)	(0.78)	(0.86)	(0.87)
Migrated out of country	0.56**	0.54**	0.47	0.66***	0.64***	0.71***
	(0.27)	(0.27)	(0.30)	(0.17)	(0.17)	(0.21)
Female		0.07	0.08		−0.26***	−0.26***
		(0.17)	(0.17)		(0.06)	(0.06)
Age		−0.07	−0.07		−0.03	−0.03
		(0.06)	(0.06)		(0.02)	(0.02)
Age_sq		0.00*	0.00*		0.00*	0.00
		(0.00)	(0.00)		(0.00)	(0.00)
High education		0.72***	0.71***		0.23***	0.23***
		(0.19)	(0.19)		(0.07)	(0.07)
Low education		0.08	0.07		−0.31	−0.30
		(0.61)	(0.61)		(0.25)	(0.25)
Log firm size		0.32***	0.32***		−0.01	−0.01
		(0.10)	(0.10)		(0.04)	(0.04)
Nurse		−0.27	−0.18		0.07	0.06
		(0.22)	(0.25)		(0.08)	(0.08)
Med. doctor		−0.05	−0.41		0.25***	0.28***
		(0.32)	(0.36)		(0.09)	(0.09)
Nurse*outmigration			−0.31			0.39
			(0.53)			(0.51)
Med. doctor*outmigration			1.71**			−0.54
			(0.78)			(0.41)
Year controlled 2006–2014	Yes	Yes	Yes	Yes	Yes	Yes
*R*	0.198	0.305	0.316	0.176	0.205	0.206
Number	841	841	841	6095	6095	6095

Source: WageIndicator 2006–2014, selection health workers born in four African countries (Angola, Kenya, South Africa and Zimbabwe) and in five Latin American countries (Argentina, Brazil, Chile, Colombia and Mexico). Reference categories: middle education, all other healthcare occupations

*Significant at 10 %; **significant at 5 %; ***significant at 1 %

In sum, concerning whether the most qualified workers migrate out of the country, the findings are mixed for the African and the Latin American countries. Even though no firm conclusions can be drawn from these analyses, the findings indicate that in the African countries, nurses as well as doctors more often out-migrate in comparison to other health workers, whereas this is the case only for doctors in the Latin American countries.

Concerning the drivers of migration, there is clear evidence that migration indeed pays off. The analyses have shown that in both country groups, out-migrated workers earn more and work fewer hours in the destination country compared to those who remained in the source country. These findings reflect the fact that median wages are higher and working hours shorter in developed countries compared to developing countries [53]. The findings show that out-migrated workers reported a higher level of life satisfaction compared to the healthcare workers who remained in the country. In the framework of these analyses, however, we cannot draw firm conclusions with respect to the drivers related to the labour market tightness in the source or destination countries. This is due to data limitations, which hinder our ability to draw any information about labour supply relative to demand. Hence, when interpreting the above results, it may well be that labour market scarcity in the host countries is a main underlying driver or that, vice versa, this could be a result of oversupply of labour in the home countries.

### Working life outcomes of migrants in destination countries

To examine whether migrant health workers are discriminated against in the destination countries compared to native health workers, we explored three types of discrimination, namely whether migrants work in occupations with a lower status, whether they work for lower wages and whether they have a lower level of life satisfaction. As indicated in the ‘Operationalization and analytical strategy’ section, we applied several multilevel random intercept models using the same variable as in the other analyses. In model 1, we first tested whether we could detect differences between migrants and non-migrants in the host countries, while model 2 controlled for several sociodemographic characteristics to test whether the ‘migration effect’ remains. In model 3, we established whether a particular group of health workers (nurses or doctors) are affected by discrimination by testing an interaction model.

Starting with the first set of models, we examined whether migrants are more likely than native workers to suffer occupational downward mobility. Table 7 shows that, contrary to our expectations, migrant health workers have a slightly higher occupational status than native workers (model 1). However, once controlled for personal characteristics, the effect becomes smaller and non-significant (model 2) and is mainly explained by higher education and medical doctor status. With respect to the interaction between being a migrant and particular health occupations, no significant effect was found for migrants in these occupational groups.

**Table 7:** Multilevel random intercept analysis for socio-economic status of occupation, log hourly wage and life satisfaction, standard errors in brackets

	Socio-economic status of occupation			Log hourly wage in PPP			Satisfaction with life		
	M1	M2	M3	M1	M2	M3	M1	M2	M3
(Constant)	59.02***	52.97***	52.97***	2.14***	0.70***	0.70***	6.44***	7.34***	7.34***
	(1.00)	(0.91)	(0.91)	(0.11)	(0.13)	(0.13)	(0.17)	(0.23)	(0.23)
Migrant	1.60***	0.16	0.24	0.06***	0.03*	0.05**	−0.09*	−0.08*	−0.15***
	(0.34)	(0.23)	(0.28)	(0.02)	(0.01)	(0.02)	(0.04)	(0.04)	(0.05)
Female		−2.66***	−2.66***		−0.13***	−0.13***		−0.05	−0.05
		(0.13)	(0.13)		(0.008)	(0.008)		(0.02)	(0.02)
Age		−0.04	−0.04		0.05***	0.05***		−0.06***	−0.06***
		(0.04)	(0.04)		(0.003)	(0.003)		(0.008)	(0.008)
Age_sq		0.0003	0.0003		−0.0005***	−0.0005***		0.0007***	0.0007***
		(0.0005)	(0.0005)		(0.00)	(0.00)		(0.00)	(0.00)
High education		8.22***	8.22***		0.27***	0.27***		0.21***	0.21***
		(0.14)	(0.14)		(0.009)	(0.009)		(0.03)	(0.03)
Low education		−3.27***	−3.27***		−0.13***	−0.13***		−0.10***	−0.10**
		(0.19)	(0.19)		(0.01)	(0.01)		(0.04)	(0.04)
Log firm size		−0.39***	−0.38***		0.07***	0.07***		0.05***	0.05***
		(0.08)	(0.08)		(0.005)	(0.005)		(0.02)	(0.02)
Nurse		−7.96***	−7.95***		−0.01	−0.006		−0.05**	−0.06**
		(0.14)	(0.15)		(0.009)	(0.009)		(0.03)	(0.03)
Med. doctor		32.45***	32.49***		0.32***	0.32***		0.12***	0.10**
		(0.22)	(0.23)		(0.01)	(0.01)		(0.04)	(0.04)
Migrant*Nurse			−0.14			−0.07*			0.12
			(0.53)			(0.03)			(0.10)
Migrant*Med. doctor			−0.41			−0.03			0.26*
			(0.71)			(0.04)			(0.13)
Survey year (2006–2014)	Yes	Yes	Yes	Yes	Yes	Yes	Yes	Yes	Yes
Sigma_u	4.97 (0.66)	1.88 (0.26)	1.88 (0.26)	0.62 (0.08)	0.65 (0.08)	0.65 (0.08)	0.91 (0.12)	0.94 (0.12)	0.94 (0.12)
Sigma_e	15.78 (0.06)	10.50 (0.04)	10.50 (0.04)	0.69 (0.003)	0.64 (0.002)	0.64 (0.002)	1.93 (0.007)	1.92 (0.007)	1.92 (0.007)
Rho	0.09 (0.02)	0.03 (0.008)	0.03 (0.008)	0.45 (0.06)	0.51 (0.06)	0.51 (0.06)	0.18 (0.04)	0.19 (0.04)	0.19 (0.04)

Source: WageIndicator 2006–2014, selection healthcare workers, *N* = 33,765 (individual level), *N* = 31 (country level), reference categories: middle education, all other healthcare occupations; all models are controlled for survey year (2006 as the ref. category)

*Significant at 10 %; **significant at 5 %; ***significant at 1 %

Regarding whether migrants receive lower wages than native workers, the results are rather surprising, as there seems to be a small wage premium for migrant health workers (model 1). Again, this effect is reduced once it is controlled for personal characteristics, but it remains significant (model 2). In addition, model 2 shows a negative wage effect for females and low educated workers, whereas a positive wage effect can be observed for age, higher education, organization size and being a doctor. Finally, testing whether particular migrant health workers receive lower wages, the interaction model (model 3) shows that the positive migration premium is significantly reduced for migrant nurses.

We were also interested in whether migrants experience a lower level of life satisfaction in comparison to native workers; the results shown in Table 7 confirmed our expectations. Model 1 shows that migrants experience on average a lower level of life satisfaction. This effect remains after including relevant personal characteristics (model 2). In addition, this model also indicates that independent of migration, older people, nurses and those who are lower educated reported being less satisfied with their lives. In contrast, being higher educated, working in a larger organization or being a doctor increases the level of life satisfaction. No significant effect was found for gender. In the last model, we again tested whether the negative life satisfaction effect for migrants differs for particular groups of healthcare occupations. The results of model 3 show that this is indeed the case for doctors, who feel a reduced negative ‘migrant effect’.

In sum, these analyses have provided some interesting insights into the labour market situation of migrant health workers in host countries. Contrary to our expectations, we did not detect discrimination against migrants with respect to wages and occupational status. In fact, there seems to be a small wage premium for the group of migrants in other healthcare occupations, which is smaller in the case of migrant nurses. The only obvious drawback to being a migrant health worker is a lower level of life satisfaction. However, this does not apply to all groups of migrants, as doctors reported a slightly higher level of life satisfaction.

## Discussion and conclusions

This study focused on the global migration patterns of health workers, what drove them to migrate and the discriminatory practices they encountered. The aim was to establish whether the WageIndicator dataset could help us answer the question whether migration actually pays off.

In this context, three research questions were addressed. In order to understand the migration patterns of health workers (RQ1), we analysed whether migration is mainly shaped by language matches, neighbouring countries or former colonial relationships. Our findings confirm existing literature suggesting that migration is indeed related to the above factors. The majority of migrants (57 %) had migrated to a country where the same language is spoken, a third (33 %) had migrated to a neighbouring country and one in five (21 %) had migrated to a former colonizing country. We also found that whereas female migrants tend to move to neighbouring countries, age only has a significant effect on having migrated to former colonizing countries, indicating that older migrants are more likely to move to former colonizing countries. This outcome may be only partly explained by the proposition made by Bach [7, 41], namely that globalization is loosening traditional ties for younger generations. Because our results indicate that particularly higher education matters for migration, we observe that highly educated migrants are more likely to have migrated to countries that have a language match rather than to neighbouring countries. We believe that this pattern points at the larger number of migratory options that more highly educated people enjoy—options that go beyond the relatively easier to access established routes of neighbouring countries, or even former colonizing countries. This is also reflected in our finding that with respect to the health workforce, nurses are more likely to migrate to neighbouring countries and to former colonizing countries, and not so much to countries where the same language is spoken, whereas doctors are more likely to migrate to countries that have a language match. This suggests that not all migratory routes are of equal status.

In order to better understand the underlying drivers of migration and whether migration pays off (RQ2), we first examined who is actually migrating. We did so by comparing the personal characteristics of migrants with those of workers in their respective source countries. Second, we looked at whether migration pays off by comparing migrants’ wages, working hours and level of life satisfaction with those of workers in the respective source countries. To answer these questions, we focused on a selected set of countries for which we had enough observations, namely four African and five Latin American countries. With respect to the question ‘Who actually migrates?’, the findings are less outspoken and mixed for the different country groups. Overall, the results show that in the African countries, nurses more often out-migrate in comparison to other health workers, whereas this is the case only for doctors in the Latin American countries. Contrary to some studies, we did not find evidence that those who migrate are mostly male [10, 38]. Instead, we found that in the Latin American countries they are more likely to be female. Concerning the pay off of migration, we found clear positive evidence. In both country groups, out-migrated workers earn more than their peers who remained in the home country, and they also work fewer hours in the host country. For the Latin American countries, it was also found that out-migrated workers reported a higher level of life satisfaction compared to the healthcare workers who remained in the country. Considering the difference in levels of economic development between these two regions, the more positive result for Latin American countries does not contradict the suggestion made by some scholars that the benefits migrants accrue may be relative to the level of development of the source countries [36, 39].

Finally, to complete the picture of the working and living condition of migrants, we also explored whether migrant health workers faced discrimination in comparison to native workers in terms of lower occupational status and wages in their destination countries, and whether they were less satisfied with their lives (RQ3). Our analyses provided some interesting insights. Contrary to our expectations that only the most elite migrants end up having a positive experience, we did not detect any forms of discrimination against migrants with respect to wages and occupational status. In fact, there seems to be a small wage premium for the group of migrants in other healthcare occupations. However, we also found that this premium is significantly less for migrant nurses. Furthermore, an obvious drawback to being a migrant health worker is the reported lower level of life satisfaction. That this outcome does not apply to all groups of migrants to the same extent (particularly migrant doctors report having a higher level of life satisfaction) does not allay the concern in qualitative literature regarding the challenges faced by nurses in destination countries. In fact, these findings point at an important difference in impact on both wage premiums and quality of life between these two cadres.

Overall, these findings lead to the conclusion that when looking at the WageIndicator dataset, the chances of migrating appear higher through care chains, including neighbouring countries with colonial and linguistic ties. Moreover, migration does seem to pay off in terms of work and labour conditions: in comparison with their non-migratory peers, migrants have higher wages and shorter working hours. Of importance is that we did not find overall evidence of downward occupational mobility and wage discrimination, although we did see a lower impact on nurses than on doctors.

Although these results are generally positive, we are aware of the differences in the accrued benefits between cadres, in particular the higher benefits accrued by doctors compared to nurses, and by migrants from more developed Latin American countries compared to those from African countries. These differences are also reflected in the different migratory routes taken. There is thus a need for more research, for instance on the impact of cultural and language competence. For example, to what extent are these patterns found in other regions of the world? And how do different migratory routes influence patterns of differentiation?

Finally, our results might be related to the characteristics of the dataset and the migrants represented therein. First, we could not model positive selectivity in this study, and this is a limitation. After all, an obvious explanation for the positive findings on wages and benefits may be related to the selective process of international migration. After all, doctors who go abroad to work speak foreign languages better, and they may be the best in their professions. Another limitation of this study is that it focused only on foreign-born migrant health workers, to the exclusion of other migrants who may be of equal relevance to the questions asked, in particular health workers born in the country of survey yet foreign trained or with a foreign or dual nationality. Both of these categories may to some extent overlap with the category of foreign-born respondents in this study. Moreover, we were not able to establish the individual participants’ length of residence in the country of birth. We do, however, believe that the category ‘foreign-born’ is one of the strongest distinguishing markers of difference among these three categories. Because of these limitations, our findings should be regarded as exploratory rather than representative.

## Additional files

Additional file 1: Distribution of 20 health care occupations over the total sample. (DOCX 17 kb) Additional file 2: a: Sample size by country, percentage migrants among health workers and most frequently mentioned migrants’ source countries. b: Sample size by year of survey. (DOCX 24 kb) Additional file 3: Job titles classified as DOCTORS and job titles classified as NURSES with sample sizes. (DOCX 15 kb) Additional file 4: Means of personal characteristics of health workers born in 4 African countries and 5 Latin American countries. (DOCX 18 kb)
