# Supplementary material for: Does migration ‘pay off’ for foreign-born migrant health workers? An exploratory analysis using the global WageIndicator dataset
Source: Hum Resour Health. 2016 Jun 24;14:40. doi: 10.1186/s12960-016-0136-5 (PMC4920982; doi:10.1186/s12960-016-0136-5)
Supplement: Additional file 1: — Distribution of 20 health care occupations over the total sample. (DOCX 17 kb) [file 12960_2016_136_MOESM1_ESM.docx]

#### Additional_file_1: Distribution of 20 health care occupations over the total sample

|  | **Percent** | **Frequency** |
| --- | --- | --- |
| Medical Doctors | 9.3 | 4122 |
| Nursing & Midwifery Professionals | 12.9 | 5721 |
| Traditional & Complementary Medicine (Associate) Profess. | 0.1 | 51 |
| Paramedical Practitioners | 0.9 | 413 |
| Veterinary Professionals | 0.1 | 35 |
| Dentists | 1.5 | 651 |
| Pharmacists | 0.9 | 410 |
| Environmental and Occup. Health and Hygiene Professionals | 1.1 | 486 |
| Physiotherapists | 3.3 | 1483 |
| Optometrists and Ophthalmic Opticians | 0.2 | 103 |
| Other Health Professionals | 11.7 | 5180 |
| Medical and Pharmaceutical Technicians | 5.9 | 2617 |
| Nurses & Midwifery Associate Professionals | 10.0 | 4422 |
| Community Health Workers | 2.5 | 1121 |
| Other Health Associate Professionals | 16.8 | 7448 |
| Personal Care Workers in Health Services | 5.6 | 2475 |
| Health Researchers & Educators | 5.2 | 2297 |
| Health Care Managers | 2.8 | 1221 |
| Health Care Administration & Operations | 5.4 | 2383 |
| Health Informatics Technicians | 4.0 | 1755 |
| Total | 100.0 | 44394 |

Source: WageIndicator 2006-2014, selection health workers, N = 44394
