# Supplementary material for: Does migration ‘pay off’ for foreign-born migrant health workers? An exploratory analysis using the global WageIndicator dataset
Source: Hum Resour Health. 2016 Jun 24;14:40. doi: 10.1186/s12960-016-0136-5 (PMC4920982; doi:10.1186/s12960-016-0136-5)
Supplement: Additional file 2: — a: Sample size by country, percentage migrants among health workers and most frequently mentioned migrants’ source countries. b: Sample size by year of survey. (DOCX 24 kb) [file 12960_2016_136_MOESM2_ESM.docx]

#### Additional_file_2a: Sample size by country, percentage migrants among health workers and most frequently mentioned migrants’ source countries

| **Country of residence** | **N** | **% migrants** | **Rank** | **Migrant country_1** | **Migrant country_2** | **Migrant country_3** | **Migrant country_4** | **Migrant country_5** |
| --- | --- | --- | --- | --- | --- | --- | --- | --- |
| Angola | 74 | 54% | 1 | Portugal | Brazil |  |  |  |
| Argentina | 1699 | 5% | 22 | Bolivia | Uruguay | Chile | Peru | Brazil |
| Azerbaijan | 157 | 18% | 7 | Russian Fed. | Armenia | Uzbekistan | Kazakhstan |  |
| Belarus | 2469 | 13% | 11 | Russian Fed. | Ukraine | Kazakhstan | Germany | Uzbekistan |
| Belgium | 2244 | 8% | 16 | Netherlands | France | Germany | Romania | Congo, Rep. |
| Brazil | 2798 | 1% | 29 | Portugal | Uruguay |  |  |  |
| Chile | 522 | 6% | 20 | Colombia | Ecuador | Uruguay | Argentina | Cuba |
| China | 69 | 0% | 34 |  |  |  |  |  |
| Colombia | 479 | 1% | 29 |  |  |  |  |  |
| Czech Republic | 960 | 4% | 23 | Slovakia |  |  |  |  |
| Denmark | 59 | 19% | 5 | Germany |  |  |  |  |
| Egypt | 96 | 2% | 25 |  |  |  |  |  |
| Estonia | 81 | 0% | 34 |  |  |  |  |  |
| Finland | 1125 | 2% | 25 | Sweden | Estonia |  |  |  |
| Germany | 8329 | 6% | 20 | Poland | Russian Fed. | Austria | Romania | Kazakhstan |
| Guatemala | 60 | 2% | 25 |  |  |  |  |  |
| Hungary | 546 | 4% | 23 | Romania |  |  |  |  |
| Indonesia | 369 | 1% | 29 |  |  |  |  |  |
| Italy | 124 | 11% | 12 | Brazil | Belgium | Romania |  |  |
| Kazakhstan | 1084 | 10% | 13 | Russian Fed. | Uzbekistan | Ukraine | Kyrgyzstan |  |
| Kenya | 68 | 19% | 5 |  |  |  |  |  |
| Korea, Rep. | 125 | 1% | 29 |  |  |  |  |  |
| Mexico | 866 | 2% | 25 | Colombia | Spain | Cuba |  |  |
| Mozambique | 120 | 35% | 2 | Portugal | Brazil | Angola |  |  |
| Netherlands | 11337 | 8% | 16 | Surinam | Belgium | NL Antilles | Germany | Morocco |
| Paraguay | 183 | 14% | 10 | Argentina | Colombia | Cuba | Spain |  |
| Poland | 426 | 0% | 34 |  |  |  |  |  |
| Portugal | 122 | 16% | 8 |  |  |  |  |  |
| Russian Federation | 935 | 8% | 16 | Kazakhstan | Ukraine | Uzbekistan | Belarus | Moldova |
| Slovakia | 272 | 1% | 29 |  |  |  |  |  |
| South Africa | 729 | 9% | 14 | Zimbabwe | UK | India | Netherlands | Botswana |
| Spain | 1253 | 9% | 14 | Argentina | Colombia | Germany | Chile | Peru |
| Sweden | 188 | 16% | 8 | Finland | Germany | USA | Iran | Bosnia and Herzegovina |
| Ukraine | 2214 | 8% | 16 | Russian Fed. | Kazakhstan | Uzbekistan | Belarus | Georgia |
| UK | 1462 | 24% | 3 | South Africa | USA | Poland | Zimbabwe | Australia |
| United States | 750 | 20% | 4 | UK | India | Canada | Germany |  |
| **Total** | **44394** | **8%** |  |  |  |  |  |  |

Source: WageIndicator 2006-2014, selection health workers, N = 44394

Additional_file_2b: Sample size by year of survey

| **Year of survey** | **Frequency** | **Percent** |
| --- | --- | --- |
| 2006 | 2066 | 4.7 |
| 2007 | 5881 | 13.2 |
| 2008 | 7663 | 17.3 |
| 2009 | 4287 | 9.7 |
| 2010 | 5153 | 11.6 |
| 2011 | 5381 | 12.1 |
| 2012 | 6266 | 14.1 |
| 2013 | 4268 | 9.6 |
| 2014 | 3429 | 7.7 |
| **Total** | **44394** | **100.0** |

Source: WageIndicator 2006-2014, selection health workers, N = 44394
