# Supplementary material for: Does migration ‘pay off’ for foreign-born migrant health workers? An exploratory analysis using the global WageIndicator dataset
Source: Hum Resour Health. 2016 Jun 24;14:40. doi: 10.1186/s12960-016-0136-5 (PMC4920982; doi:10.1186/s12960-016-0136-5)
Supplement: Additional file 3: — Job titles classified as DOCTORS and job titles classified as NURSES with sample sizes. (DOCX 15 kb) [file 12960_2016_136_MOESM3_ESM.docx]

#### Additional_file_3: Job titles classified as DOCTORS and job titles classified as NURSES with sample sizes

| **DOCTORS** |  | **NURSES** |  |
| --- | --- | --- | --- |
| General Practitioner | 841 | Charge nurse | 569 |
| Company doctor | 106 | DEU Charge nurse | 1 |
| Anaesthetist | 278 | Children’s nurse | 202 |
| Gynaecologist | 136 | District nurse | 446 |
| Psychiatrist | 190 | Hospital nurse | 2035 |
| Surgeon | 404 | DEU Hospital nurse | 3 |
| Urologist | 48 | Intensive care, recovery nurse | 407 |
| Pathologist | 46 | Psychiatric nurse | 531 |
| Gastroenterologist | 19 | Surgical nurse | 97 |
| Geneticist | 6 | Nurse, all other | 1399 |
| CZE SVK Hematologist | 1 | Professional midwife | 31 |
| Cardiologist | 85 | Nursing associate professional | 3069 |
| Skin specialist | 35 | DEU Nursing associate professional | 22 |
| Optical specialist | 44 | Company nurse | 90 |
| Plastic surgeon | 14 | Private nurse | 131 |
| Radiologist | 103 | School nurse | 79 |
| Medical practitioner, all other specialists | 1730 | Assistant midwife | 27 |
| DEU Medical practitioner, all other specialists Chefarzt | 8 | Nursing aide (clinic or hospital) | 1004 |
| DEU Medical practitioner, all other specialists Oberarzt | 26 |  |  |
| Toxicologist | 2 |  |  |

Source: WageIndicator 2006-2014, selection health workers, N = 44394
