# Supplementary material for: Does migration ‘pay off’ for foreign-born migrant health workers? An exploratory analysis using the global WageIndicator dataset
Source: Hum Resour Health. 2016 Jun 24;14:40. doi: 10.1186/s12960-016-0136-5 (PMC4920982; doi:10.1186/s12960-016-0136-5)
Supplement: Additional file 4: — Means of personal characteristics of health workers born in 4 African countries and 5 Latin American countries. (DOCX 18 kb) [file 12960_2016_136_MOESM4_ESM.docx]

#### Additional_file_4: Means of personal characteristics of health workers born in 4 African countries and 5 Latin-American countries

|  | **4 African countries** | | | **5 Latin American countries** | | |
| --- | --- | --- | --- | --- | --- | --- |
|  | **Outmigrating** | **Remained in country** |  | **Outmigrating** | **Remained in country** |  |
| Female | 62% | 68% | ns | 61% | 53% | * |
| Age (15-64) | 36.2 | 34.5 | * | 36.0 | 34.0 | ** |
| High education | 77% | 75% | ns | 76% | 73% | ns |
| Low education | 3% | 2% | ns | 5% | 1% | *** |
| Medical doctor | 11% | 6% | * | 23% | 14% | *** |
| Nurse | 29% | 14% | *** | 13% | 14% | ns |
| N | 140 | 750 |  | 181 | 6175 |  |

Source: WageIndicator 2006-2014, selection health workers born in 4 African countries and 5 Latin American countries,
N = 7,246.
